# The causes of holes and loss of physical integrity in long‐lasting insecticidal nets

**DOI:** 10.1186/s12936-020-03567-0

**Published:** 2021-01-19

**Authors:** Amy Wheldrake, Estelle Guillemois, Hamidreza Arouni, Vera Chetty, Stephen J. Russell

**Affiliations:** Nonwovens Innovation & Research Institute Ltd, 169 Meanwood Road, Leeds, LS7 1SR West Yorkshire UK

**Keywords:** Long-lasting insecticide-treated nets, Physical integrity, Durability, Resistance to damage, Holes

## Abstract

**Background:**

Long-lasting insecticidal nets (LLINs) are expected to last for at least 3 years, but whilst this may be achieved from an insecticidal perspective, physical protection is frequently compromised much earlier because of the rapid accumulation of holes during use. To understand why LLINs are so susceptible to loss of physical integrity, thousands of hole damage sites in LLINs retrieved from the field in Africa and Asia were forensically studied to identify the persistent underlying causes.

**Methods:**

A total of 525 LLINs consisting of six different brands from five different countries across Africa and Asia were collected from the field after 1 to 3 years in use. More than 42,000 individual sites of hole damage were analysed based on the morphology and size of each individual hole, aided by optical microscopy (OM) and scanning electron microscopy (SEM). The fracture morphology enabled positive identification of the underlying mechanisms of the damage.

**Results:**

Across all LLINs and geographical settings, mechanical damage is the primary cause of holes and loss of physical integrity in LLINs (63.14% by frequency and 81.52% by area). Snagging is the single most frequent mechanical damage mechanism, whilst the largest sized holes in LLINs result from seam failure and tearing. Abrasion and hole enlargement are also responsible for a progressive loss in the physical integrity of nets. Collectively, these five modes of mechanical damage can be expected to result from normal use of LLINs by households. Evidence of deliberate cutting, burn holes and rodent damage was observed to a lesser degree, which LLINs are not designed to withstand.

**Conclusions:**

Loss of physical integrity in LLINs is an inevitable consequence of using a vector control product that has an inherently low resistance to mechanical damage during normal use. To improve performance, new specifications based on laboratory textile testing is needed, to assess the resistance of LLIN products to the primary causes of mechanical damage when in use, which are snagging, tearing, abrasion and hole enlargement. Seam construction also needs to meet a revised minimum standard to reduce the risk of a rapid loss of physical integrity during use.

## Background

Long-lasting insecticidal nets (LLINs) are an established vector control tool responsible for saving many lives. According to the World Health Organization (WHO), cases of malaria fell 1.3% from 231 million in 2017 to 228 million in 2018 [1]. However, recent rises in incidence have been linked to concerns about the condition and physical integrity of LLINs after a few years of use, and the potential for heightened risk of malaria transmission during the inter-campaign time period. It is now generally regarded as ‘inevitable’ that LLINs will accumulate holes as users interact with the product following distribution. Numerous field studies report accumulation of holes within the first two years of use, with many LLINs becoming so badly damaged that they are discarded [2–7], regardless of whether they still retain insecticidal functionality. Ideally, LLINs should provide physical protection for at least three years, but it is apparent that this is not being consistently achieved.

Until now, evaluations of the physical integrity of LLINs have relied on surveying the amount of damage they incur during use, by characterizing the location, size and frequency of holes [8, 9]. It is apparent from these studies that while the relative degrees of damage may differ, all existing LLINs are susceptible to hole formation, irrespective of brand. The extent of damage in each setting is of course affected by a range of human behavioural and environmental factors, but there have been relatively few attempts to positively identify root cause mechanisms [10, 11]. Previous efforts to identify the underlying causes of damage and hole formation in LLINs have mostly relied on user questionnaires and observations in the field [12]. These studies have attributed hole formation to a variety of factors including tearing, thermal damage due to candles or cooking embers and contact with rodents.

A forensic approach to identifying root causes was introduced by Käse and Russell based on direct microscopic analysis of hole damage sites in LLINs retrieved from the field in Africa [13]. The study, which looked at both PET and PE-based LLINs revealed three major causes of holes: (i) mechanical damage (in the form of snags, abrasion and tears), (ii) thermal damage (caused by proximity to naked flames and embers) and (iii) animal damage (rodent interaction). Mechanical damage was found to be the most frequent cause of holes, with the majority of the damage being consistent with normal use of the LLIN within households. To a limited extent, there was also evidence of deliberate damage to LLINs, such as where knives had been used to cut the net fabric, possibly to improve access. Therefore, when considering the holes accumulated during use, it was possible to distinguish between ‘*reasonable use*’ and ‘*unreasonable use*’ of the vector control product. In this context, reasonable use referred to forces and damage mechanisms that would be difficult to avoid if the LLIN was used as originally intended, whereas unreasonable use (or careless use) involved situations where there was exposure to mechanisms of damage that the product was never originally designed to resist.

The Käse and Russell [14] study involved a relatively small sample of LLINs retrieved from one geographical location in Africa, and a larger study is required to determine the extent to which findings are representative. In the present work, the primary causes of hole formation leading to loss of physical integrity in LLINs in large sample of over 500 LLINs retrieved from multiple settings across Africa and Asia, comprising multiple brands were identified. Understanding why holes form so easily during use is essential if underlying causes are to be addressed by an appropriate strategy to improve the physical integrity of LLINs.

## Methods

The collection of nets from the field was overseen by Dr. A. Kilian with the kind assistance of Centers for Disease Control and Prevention (CDC), President’s Malaria Initiative (PMI), WHO Pesticide Evaluation Scheme (WHOPES) and Tropical Health. A total of 525 LLINs made of polyethylene terephthalate (PET) or polyethylene (PE) were retrieved from the field in 2013, 1 to 3 years after use, across five different countries: India, Mozambique, Nigeria, Uganda and Kenya. Nets received from Nigeria were from three separate locations. Thus, a total of seven separate samples based on geographical location were included in the study. The sample comprised 163 Permanet nets (PET multifilament), 98 Olyset nets (PE monofilament), 139 Duranet nets (PE monofilament), 47 NetProtect nets (PE monofilament), 54 Dawaplus nets (PET multifilament) and 34 Interceptor nets (PET multifilament). The time in use ranged from 12 to 36 months.

Damage sustained by polymer and textile materials as a result of mechanical forces, heat and other agencies during use result in characteristic fracture morphologies that are extensively reported in the textile science literature [15–19]. Aided by optical microscopy (OM) and scanning electron microscopy (SEM), damage morphologies in all 525 LLINs (> 40,000 holes) were individually analysed and categorized to determine root cause mechanisms. Examples of damage morphologies in LLINs and associated causes are summarized in Table 1. Holes associated with different damage mechanisms were counted and numerical frequency of their occurrence calculated.

**Table 1:** Hole damage morphologies identified in LLINs retrieved from the field

Type of damage	Damage morphology	Mechanism
Snag		A yarn is pulled or plucked from the surface of the LLIN after becoming caught on a solid pointed object
Tear		Tensile breakage of yarns within the fabric plane, in one or more directions. For example, after the LLIN is caught on a solid pointed object and then pulled in a perpendicular direction
Abrasion		Wearing away of yarns in the LLIN by rubbing against other surfaces. Broken filament ends project from the LLIN fabric surface
Cut		Yarns are sliced by a knife or blade drawn through the structure. This involves sharp transverse load and cleaved filament ends with limited distortion of the LLIN fabric structure
Thermal		Melting of the polymer in the yarn due to localised high temperature exposure. Hard, melted and/or charred filament ends and shrinkage of the adjacent LLIN fabric structure are typical
Animal (rodent)		Shredded, frayed or ragged edged yarn breakages in the LLIN fabric, often combined with discolouration, resulting from gnawing
Seam failure		Breakage of the seam between two panels of the LLIN, leading to immediate separation
Laddering		Enlargement of an initial hole by pulling out of successive knitted loops in the LLIN fabric structure following yarn breakage
Unravelling		Enlargement of an initial hole by unlooping of the yarns in the knitted LLIN fabric structure following initial yarn breakage

**Fig. 1:**
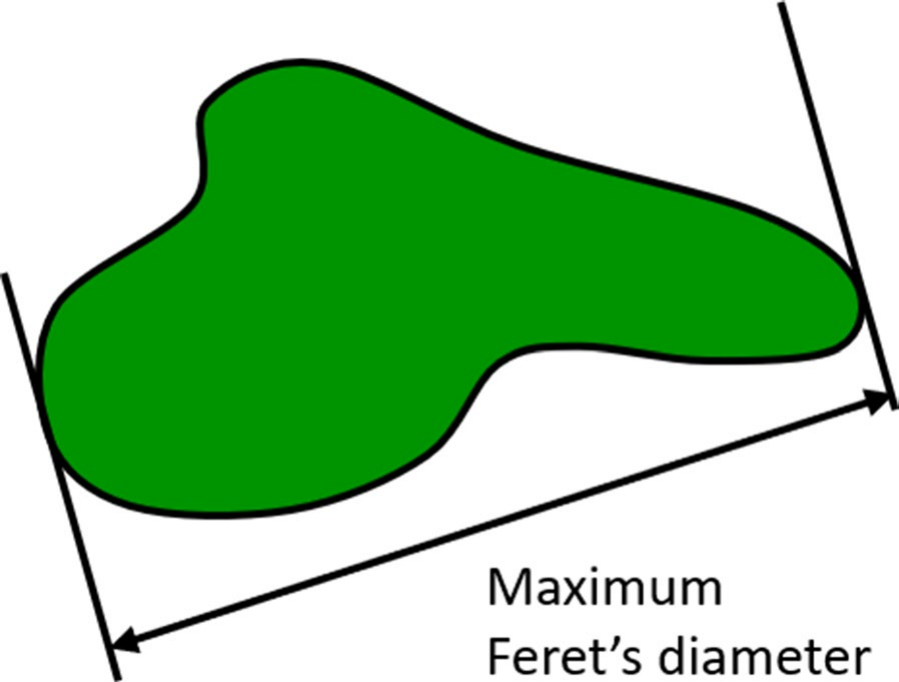
Hole size by Feret’s diameter

Hole size was also measured to explore potential linkage with specific causes of damage. The Feret diameter (Fig. 1) was recorded, i.e. the distance tangential to the boundary of the hole at its largest dimension. Note that any break in a yarn within the LLIN fabric can be regarded as a hole defect and was therefore recorded. Total hole area was calculated as the sum of all hole areas attributed to an individual damage and across all analysed holes.

WHO guidelines recommend use of the proportionate hole index (PHI) to assess net fabric integrity [20]. The hole damage areas are expressed as a proportion of the total hole area. Hole size classification was based on WHO guidelines as summarized in Table 2. The hole index is based on the hole area as defined by the WHO hole size categories defined in Table 2. The hole index is then calculated by weighting each hole by size based on the hole area, as outlined in Table 2, and summing values for each net.

**Table 2:** WHO hole size guidelines and hole index used to assess physical integrity of LLINs

WHO 2013 guidelines	Size banding	Hole diameter	Hole radius		Area of hole	Hole index^a^
	cm	d; cm	r = d/2; cm	r^2^; cm^2^	cm^2^	
Size 1 Smaller than a thumb	0.5–2	1.25	0.625	0.3906	1.23	1
Size 2 Larger than a thumb but smaller than a fist	2.5–10	6	3	9	28.28	23
Size 3 Larger than a fist but smaller than a head	11–25	17.5	8.75	76.5625	240.56	196
Size 4 Larger than a head	≥ 26	30^b^	15	225	706.95	576

A -area of the hole pr^2^; p = 3.142

^a^Area divided by 1.23

^b^Assumer diameter

Thus, if the weighting of the hole sizes 1, 2, 3 and 4 is 1, 23, 196 and 576 respectively, the hole index (H_i_) per LLIN is calculated as in Eq. 1:

1 $${\text{H}}_{i} = \left(1\times {\text{N}}_{1}\right)+\left(23 \times {\text{N}}_{2}\right)+\left(196 \times {\text{N}}_{3}\right)+\left(576 \times {\text{N}}_{4}\right)$$

where N_1_ is the number of size 1 holes, N_2_ is the number of size 2 holes, N_3_ is the number of size 3 holes and N_4_ is the number of size 4 holes.

## Results

The hole morphologies summarized in Table 1 were found to accurately represent the full range of damage types observed in retrieved LLINs irrespective of brand or retrieval location. For all LLINs, the proportion of all holes attributable to each of these damage mechanisms was determined, both in terms of numerical frequency as well as their size.

### Damage mechanisms by hole frequency

**Fig. 2:**
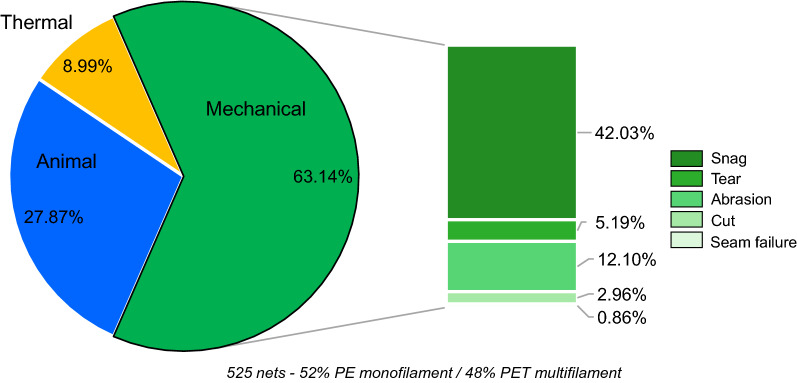
Proportion of holes by frequency in all analysed LLINs caused by the specific damage mechanisms

Holes in all LLINs resulted from combinations of mechanical, thermal or animal damage. Of these, mechanical damage consistently accounted for the majority of holes (63.14%) by frequency, followed by animal (rodent) and thermal damage at approximately 28.87% and 8.99%, respectively (Table 3). As shown in Fig. 2, the mechanical damage in LLINs could be further broken down into five recurrent mechanisms: snagging, tearing, abrasion, seam failure and cutting. Secondary damage such as laddering and unravelling were associated with hole enlargement following the initial mode of failure.

**Table 3:** Damage mechanisms ranking and proportion of hole damage by frequency in LLINs caused by damage mechanisms

Ranking of damage mechanism by hole frequency	Cause	Proportion by frequency (%)
1	Mechanical	63.14
2	Animal	27.87
3	Thermal	8.99

All LLINs were susceptible to mechanical damage with snags being by far the most frequently encountered mechanism, responsible for 42.03% of the total number of holes present (Fig. 2). The second most frequent cause of mechanical damage was abrasion (12%). Tears, seam failure and cuts accounted for less than 10% of the total damage by numerical frequency, lower than thermal damage (8.99%), and animal (rodent) damage (27.87%).

### Damage mechanisms by hole area and size

**Fig. 3:**
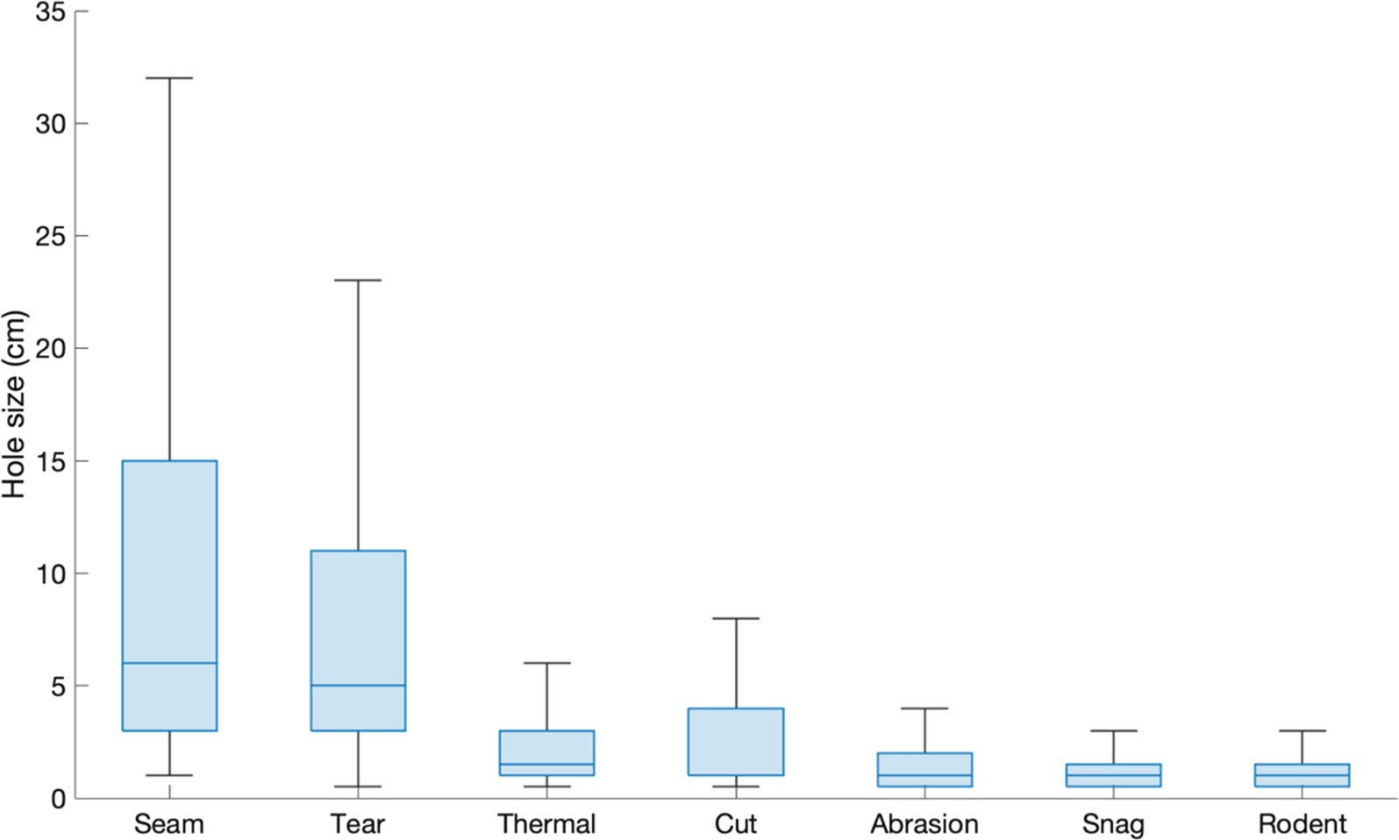
Box plot of the hole size for specific hole damage mechanisms

When considered in terms of total hole area, mechanical damage was again responsible for the largest proportional total hole area (81.5%) across all LLINs (Table 4), followed by animal (rodent) and thermal damage (10.6% and 7.9%, respectively). Interestingly, Fig. 3 reveals that the less numerically frequent forms of hole damage reported in Fig. 2, were responsible for the largest hole sizes. While tears and seam failure were relatively infrequent, compared to for example, snags (Fig. 2), these two mechanisms resulted in the largest hole areas, and produced the largest individual hole diameters, with median values of 5 cm and 6 cm respectively (Fig. 3).

**Table 4:** Damage mechanisms ranking and proportion of total hole area in LLINs caused by damage mechanisms

Ranking of damage mechanism by hole area	Cause	Proportion of total hole area (%)
1	Mechanical	81.52
2	Animal	10.63
3	Thermal	7.86

**Fig. 4:**
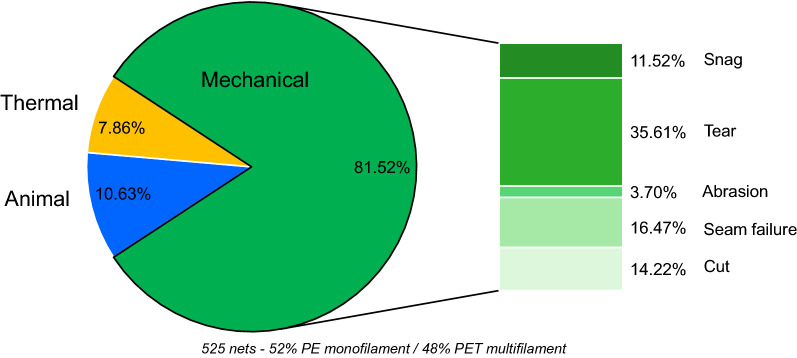
Proportion of total hole area in LLINs collected in different countries caused by damage mechanisms

Despite their high numerical frequency (Fig. 2), snag and animal (rodent) hole damage each accounted for only small proportion (about 11%) of the total hole damage by area in LLINs, while the smallest hole area was associated with abrasion (3.7%) (Fig. 4). This is linked to the fact that the most frequent holes resulting from snagging, animal damage and abrasion resulted in very small median hole sizes of only ~ 1 cm. Tearing was responsible for 35.61% of the total hole area (Fig. 4), followed by seam failure (16.47%) and cuts (14.22%). Seam failure was also associated with the largest range of hole sizes, with some reaching 15 cm in diameter (Fig. 3), so large in fact that the physical protection offered by these LLINs could be considered questionable.

As is also evident in Fig. 3, cuts and thermal damage produced relatively small holes (median = 1 cm and 1.5 cm, respectively) but there was greater size variation than with snags, rodent and abrasion hole damage.

**Fig. 5:**
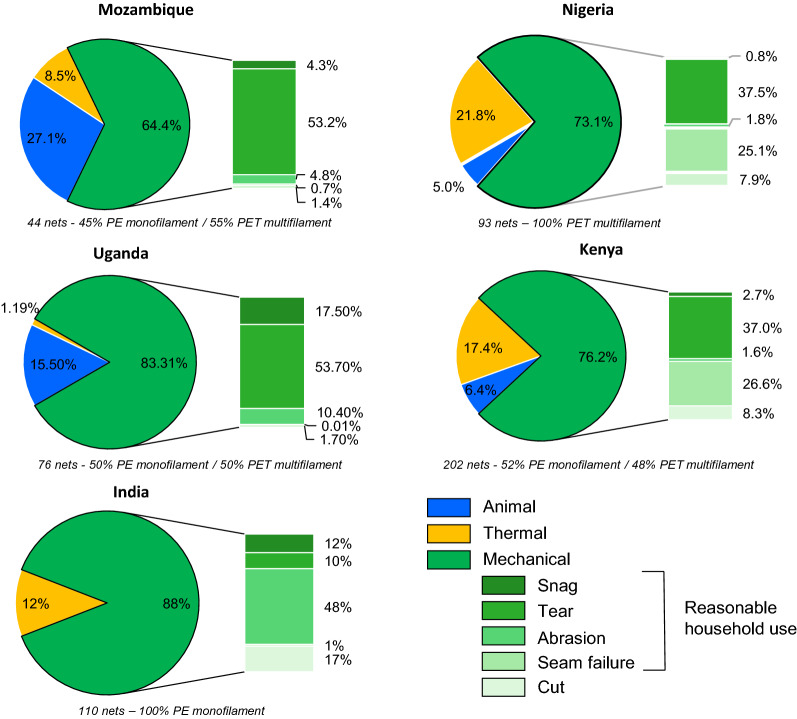
Proportion of total hole area in all analysed LLINs caused by specific damage mechanisms

The proportion of the total hole area caused by each of the damage mechanisms was further investigated with regard to specific countries. Regardless of where the LLINs were distributed and how the LLINs had been constructed, mechanical damage accounted for more than 64% of the total hole surface area for each country as illustrated in Fig. 5.

### Proportional hole index

PHI is a particularly useful indicator of the relative magnitude of LLIN damage because it is influenced by both hole size and frequency. In this study, it was possible for the first time to attribute PHI values to specific hole damage mechanisms. For this analysis, to give an insight in to inherent differences in the resistance to damage of LLIN products made in different ways, PHI values were calculated for six different LLIN products (retrieved from the same location, after the same period of use). This sample consisted of LLINs made from 48% of PET multifilament yarns and 52% of PE monofilament yarns (Table 5). To gain an insight into the inherent ability of different LLINs to withstand the agencies of wear and tear normally encountered during household use, only mechanical hole damage in the LLINs due to *reasonable use* was considered in the PHI data, i.e. snagging, tearing and abrasion. Hole formation due to seam failure was not included in the investigation as poorly constructed seams is possible irrespective of type of filament yarn and net structure.

**Table 5:** Number of holes per size category for all LLINs and mean PHI per LLIN

Filament specification and LLIN fabric knitting pattern	Number of holes per size category for all LLIN				Mean PHI per LLIN
	Size 1	Size 2	Size 3	Size 4	
All PET multifilament nets	1954	367	57	23	363
All PE monofilament nets	1083	219	51	24	282
145 Denier PE monofilament—knitting pattern 1	308	45	5	4	160 (lowest PHI)
75 D or 100D PET multifilament—knitting pattern 2	739	155	21	14	485 (highest PHI)

Table 5 reveals that LLINs made from PE monofilament yarns exhibited lower PHI values in the Size 1 and 2 categories (1083, 219) compared to PET multifilament nets (1954, 367). Referring to Fig. 3, hole sizes of 0.5–2 cm (size 1) relate to holes caused by snagging and abrasion while hole sizes of 4–8.5 cm (size 2) relate to tear damage. The marked difference in the PHI for the size 1 category, can be attributed to multifilament yarns being more prone to snagging on solid objects and abrasion, compared to those made from monofilament. The difference is therefore attributable mainly to the type of filament yarn (monofilament, multifilament) used to make the LLIN and not the polymer composition (PET, PE).

All LLINs regardless of knitting pattern and filament linear density (denier) suffered hole formation across all PHI size categories, due to mechanical damage, reflecting a progressive loss of physical integrity over a period of use. However, from Table 5 it was apparent that some LLIN products were more resistant to mechanical damage than others. Across all size ranges (PHI size 1–4), the knitting pattern 1 LLIN structure made of 145 denier PE monofilament yarns consistently produced the lowest number of holes in each size category, compared to the knitting pattern 2 structure made of 75 or 100 denier PET multifilament yarns.

### Hole enlargement

Regardless of their initial dimensions, it is known that existing holes in LLINs can enlarge into bigger ones [13] during subsequent use, producing characteristic damage morphology (Table 1). Even a single yarn breakage in a LLIN can be sufficient to seed the formation of a larger hole due to secondary damage such as laddering or unravelling of the fabric structure. Laddering and unravelling defects were observed in many of the LLINs (Table 6). Unlike tearing, unravelling does not require yarns to progressively break, but rather disassembly of the net structure occurs following an initial break of a yarn, as knitted loops slip apart. This has the capacity to produce large holes and is heavily influenced by the choice of knitting pattern used to make the LLIN.

**Table 6:** Proportion of holes exhibiting secondary damage by frequency in all analysed LLINs

Population	% of holes exhibiting unravelling and tearing	% of holes exhibiting laddering	% of ladders combined with tearing
Total across all LLINs	5.41	6.13	18.11

Consequently, small initial hole damage in LLINs such as that created by snags is important because even one yarn breakage could be sufficient to seed the formation of a larger hole later.

## Discussion

Effective, long-lasting insecticides [21–23] are essential to the future of vector control, but these efforts are likely to be seriously undermined if the physical integrity of LLINs does not markedly improve [24–26]. In this study, the primary causes of holes and thereby loss of physical integrity in LLINs were identified, so as to understand why these important vector control products are prone to rapid deterioration after only a few years of use. It was discovered that mechanical damage is the primary cause of hole formation in all LLINs both in terms of frequency (63.14%, Table 3; Fig. 2) and total hole area (81.52%, Table 4; Fig. 4), confirming the previous findings of Russell and Käse, whose findings were based on a smaller sample size, and confined to a retrieval site in South East Ghana [13].

Although mechanical damage caused by snagging produced small holes (Fig. 3), it was by far the most frequent cause of damage in all LLINs (Fig. 2), highlighting the high susceptibility of net fabrics to this form of physical deterioration. Snagging is a well-known deficiency of lightweight knitted fabrics, which are now typically less than 55 g/m^2^ [27], and damage can only be expected to worsen if LLIN fabric weights decrease. LLIN fabrics made of multifilament yarns were more prone to snagging than those made from monofilaments, which had the effect of increasing the PHI due to the frequency of holes in the size 1 category (Table 5). As in the case of mechanical damage in the form of snagging, abrasion resulted in relatively small holes ranging in size from 0.5 to 2 cm (Fig. 3). This results from progressive abrasive wear of the yarns in the LLIN fabric as they are rubbed against a solid surface resulting ultimately in yarn breakage. Although the small holes that snagging and abrasion produces may not initially compromise the physical protection provided by the LLIN, there is a risk of hole enlargement during subsequent use, as confirmed by Table 6, due to unravelling or laddering.

The largest holes found in LLINs were caused by seam failure and tearing (Fig. 3), both of which are forms of mechanical damage. Together these two mechanisms accounted for over 50% of the total hole area in LLINs (Fig. 4). Seam failure in this context primarily results from poor manufacturing practice, because in certain seam constructions, the breakage of just one yarn within the seam can lead to rapid separation of the LLIN panels, rendering it essentially useless in terms of physical protection. Seam failure can be largely mitigated by ensuring LLINs are made with appropriate seam constructions in the first place. Tearing also produces large holes and accounted for more than 35% of the total hole area in LLINs (Table 4). Tears usually form as a result of the net first being snagged on a solid object, such as wooden mattress material, and then when force is applied to pull it free, tearing is induced as individual yarns break. Polymer, yarn and fabric properties, as well as the basis weight of the LLIN fabric will all influence the susceptibility of the LLIN fabric to tearing.

Efforts to limit the rate of physical deterioration of LLINs depend on future products having significantly improved resistance to the mechanical damage during reasonable use in the household and/or being used more carefully. Clearly, the former depends on improved LLIN product design and appropriate technical specifications, and the latter depends on behavioural change. Behavioural change will require promoting careful deployment, utilisation and storage of LLINs by users (primarily), regular repair and maintenance and keeping nets clean to reduce the need for washing [28]. Keeping nets away from candles, cooking embers and cigarettes, as well as preventing their misuse for purposes, such as food storage, fishing or other means of use different from the intended vector protection is also important. However, more careful use, is unlikely to be sufficient on its own to address the challenge, unless the basic ‘resistance to damage’ of current LLIN vector control products is significantly improved. Even if LLINs are used relatively carefully, mechanical damage is highly likely to accumulate, and rate of attritional wear would be expected to be greater if there are children in the household or nets are not looked after properly [10].

Given the real-world conditions that LLINs and users face, more physically robust LLINs are needed to reduce hole formation and enable significant improvements in long-term physical integrity and durability to be achieved. This means designing and specifying LLIN vector control products that are better able to cope with the normal agencies of mechanical wear and tear they will encounter during reasonable household use. Specifically, this means designing improved LLIN products with the capability to resist snagging, tearing, seam failure, abrasion and hole enlargement. For economic reasons alone, it would be highly challenging to design cost-effective LLIN products with the ability to resist unreasonable use resulting from cutting, rodent interactions or high temperature thermal damage. However, a combined approach of implementing products with higher inherent resistance to mechanical damage, together with behavioural change measures, is likely to be an effective strategy for improving the long-term physical integrity of LLINs.

It is apparent from the present work that LLINs are subjected to a group of forces during normal household use, that are not reflected in product specifications prior to distribution. Currently, the only routine laboratory test conducted on LLINs to evaluate their resistance to damage is bursting strength, which as a sole measure of mechanical strength, does not fully reflect the real mechanisms by which LLINs are deteriorating in users’ households. Of the mechanical damage observed in LLINs associated with reasonable use of the product, it is believed that approximately 80% is preventable by improved design and specification of the LLIN itself. Resistance to snagging, tearing, abrasion, seam failure and hole enlargement can all be improved and should be tested in the laboratory to make sure the physical condition of LLINs is less susceptible to deterioration before distribution to vulnerable users.

## Conclusions

Mechanical damage is the primary cause of hole formation and loss of physical integrity in LLINs, whether expressed in terms of frequency or area (63.14% and 81.52%, respectively). Regardless of where the LLINs were distributed, mechanical damage accounted for more than 64% of the total hole area in LLINs. This relates to a group of damage mechanisms that LLINs are exposed to during normal household use: snagging, tearing, abrasion and hole enlargement. Increasing the inherent resistance of LLINs to these specific forms of mechanical damage is likely to substantially reduce their susceptibility to hole formation, thereby improving long-term physical integrity. The largest holes in LLINs are attributable to tearing and seam failure, the latter being indicative of poor manufacturing practice, but small holes should also not be ignored because of their potential to enlarge over time. To increase physical integrity, the inherent resistance to mechanical damage of LLIN vector control product needs to be improved. Seam construction requires a new minimum standard, and all LLIN fabrics should be subjected to a suite of textile tests to evaluate and specify their snag strength, tear strength, abrasion resistance and resistance to hole enlargement.
